# Effects of skin tone and adipose thickness on frequency domain near-infrared spectroscopy and diffuse correlation spectroscopy

**DOI:** 10.1117/1.BIOS.2.1.012503

**Published:** 2024-12-06

**Authors:** Carlos A. Gómez, Darren Roblyer

**Affiliations:** Boston University, Department of Biomedical Engineering, Boston, Massachusetts, United States

**Keywords:** adipose, diffuse correlation spectroscopy, frequency-domain diffuse optics, multi-layer models, near-infrared spectroscopy, skin tone

## Abstract

**Significance:**

Frequency domain near-infrared spectroscopy (FD-NIRS) and diffuse correlation spectroscopy (DCS) are used for the label-free measurement of chromophore concentrations, blood flow, and metabolism for tissues such as muscle or tumors. These tissues are embedded under the skin and adipose, the properties of which can vary between subjects, thus affecting the extraction of the target tissue’s optical properties.

**Aim:**

We aim to characterize the effects of the skin tone and adipose thickness on FD-NIRS and DCS measurements and develop subject-specific multi-layer inverse models that account for these effects.

**Approach:**

A three-layer look-up-table-based inverse model that accounted for the skin tone and adipose thickness was generated using Monte Carlo simulations for each subject. Stackable tissue-mimicking silicone phantoms were fabricated and used to validate the models. A custom combined FD-NIRS and DCS system was then used to measure phantoms and the sternocleidomastoid muscle of healthy subjects. Subjects performed a breathing exercise that consisted of a baseline, load, and recovery. The skin tone of subjects was determined using a colorimeter. The adipose thickness was determined using ultrasound. The subject-specific three-layer model results were compared against a simpler single-layer model.

**Results:**

The skin tone and adipose thickness substantially affected the extraction of multiple FD-NIRS and DCS parameters. Oxygenated hemoglobin, total hemoglobin, tissue saturation ($S_{t}O_{2}$), and blood flow index (${\mathrm{BF}}_{i}$) values were all underestimated if the skin tone and adipose thickness were not accounted for (all p-values<0.01). For example, $S_{t}O_{2}$ was underestimated by 18±9 %pt (p<0.0001) and ${\mathrm{BF}}_{i}$ was underestimated by $7\pm 8\times 10^{-6}\;{\mathrm{mm}}^{2}/s$ (p<0.01). Hemodynamics during a respiratory exercise were also underestimated in the case of oxygenated hemoglobin, total hemoglobin, ${\mathrm{BF}}_{i}$, and metabolic rate of oxygenation (all p<0.05).

**Conclusion:**

We highlight the importance of accounting for both adipose thickness and skin tone when targeting underlying tissue. The multi-layer models we developed have the potential to be applied to a wide range of *in vivo* studies.

Statement of Discovery We discovered that skin tone and adipose layer thickness had a dramatic effect on the extraction of hemoglobin concentrations, oxygen saturation, blood flow, and metabolic rate of oxygen consumption. The subject-specific multi-layer models developed here, which take into account both skin tone and adipose thickness, could lead to more accurate *in-vivo* measurements across diverse subject populations when using frequency domain near infrared spectroscopy and diffuse correlation spectroscopy.

## 1 Introduction

Frequency domain near-infrared spectroscopy (FD-NIRS) and diffuse correlation spectroscopy (DCS) are powerful tools that use near-infrared light to provide continuous label-free monitoring of tissue. FD-NIRS provides quantitative measurements of tissue optical properties, which can be used to recover absolute chromophores concentration such as hemoglobin, lipid, and water; meanwhile, DCS provides blood flow information of the tissue. Together, FD-NIRS and DCS can provide the metabolic rate of oxygen consumption. These methods have been used either individually, or together, to measure the muscle tissue, tumors, and brain tissue over a wide variety of basic science and clinical applications.^1^^–^^7^ These techniques are often used to measure a target tissue (e.g., muscle, tumor, and brain) that is embedded under overlying tissue layers that might include the skin, adipose, and bone. Despite the fact that the optical properties and thickness of these layers can vary widely across subjects, it is still common to use simple inverse models that assume the measured medium is homogeneous.^8^ This is because knowledge of the overlying tissues is often limited in practice. Measurements at multiple source–detector separations are often used to account for superficial versus deeper tissue,^9^^–^^12^ which can improve results. There is an increasing recognition that overly generalized assumptions about individual features such as the skin tone can lead to erroneous or misleading results that may lead to an incorrect clinical course of action for optical techniques. An important case study is the use of pulse oximetry during the COVID-19 pandemic. Studies conducted during this time led to the recognition that patients with darker skin were commonly misdiagnosed as having peripheral oxygen saturation levels in a healthy range when they were in fact hypoxic.^13^ Recent work in the photo-acoustic community has shown that darker skin tone leads to an overestimation of blood oxygen saturation.^14^ Other studies have recently found that adipose thickness could substantially affect photoplethysmography (PPG) measurements.^15^^,^^16^ These studies highlight the need for subject-specific models for techniques such as FD-NIRS and DCS.

Several prior works have developed inverse models for FD-NIRS and DCS that utilize either analytical solutions or Monte-Carlo-based look-up tables to account for multiple layers in tissue.^17^^,^^18^ The neurophotonics community has leveraged multi-layer models to account for the effects that the skull has on brain measurements for both FD-NIRS and DCS measurements.^19^^–^^21^ In addition, it has been shown that accounting for subcutaneous tissue (skin plus adipose) during DCS measurements improves the accuracy of muscle blood flow index (${\mathrm{BF}}_{i}$).^9^ Despite these prior works, there has been minimal exploration of the combined effect of the skin tone and adipose thickness on FD-NIRS and DCS measurements.

We present here a Monte-Carlo-based processing pipeline that allows for the creation of subject-specific multi-layer LUTs that take into account both skin tone and adipose thickness to more accurately recover target tissue properties. We developed a set of LUT-based inverse models that span skin tones from light, medium, to dark, and adipose thicknesses up to 6 mm in increments of 1 mm. Although somewhat computationally expensive to generate, these LUTs provide rapid inversions that can be used across a wide range of skin and body phenotypes. We then validated the accuracy of these models using custom stackable multi-layer phantoms. We also utilized the models in a healthy volunteer study and showed that taking into account the skin and adipose led to significant changes in extracted optical properties and chromophore concentrations. Looking forward, subject-specific models such as these may help to improve accuracy and prevent misdiagnosis across many clinical applications.

## 2 Methods

### 2.1 Monte-Carlo-Based Multi-Layer Inverse Models

**Fig. 1:**
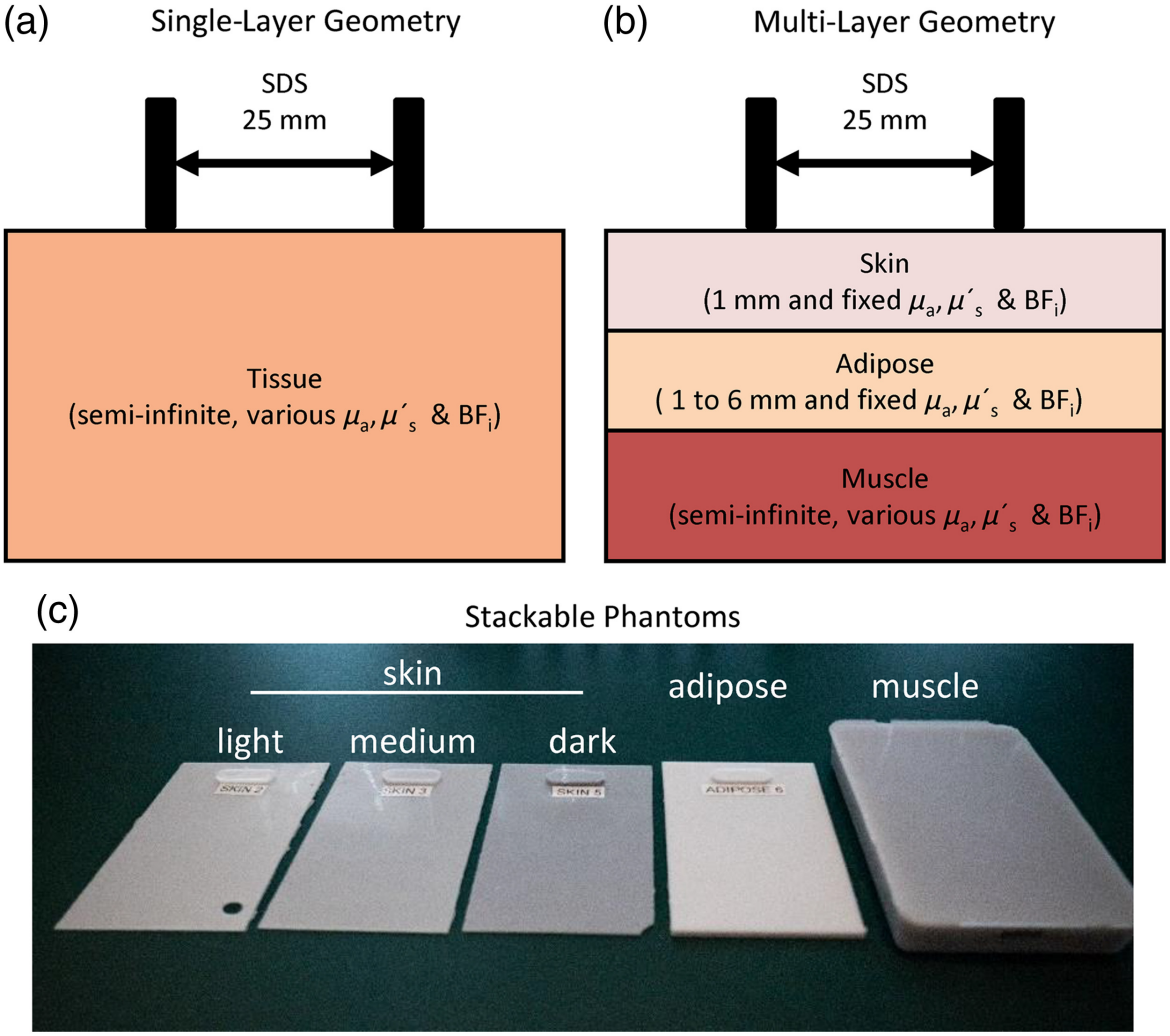
(a) Monte Carlo geometry used in single-layer models. (b) Monte Carlo geometry used in multi-layer models. (c) Stackable phantoms used in phantom measurement. From left to right: light skin, medium skin, dark skin, adipose tissue, and muscle. SDS, source–detector separation; $\mu_{a}$, absorption coefficient; $\mu_{s}^{\prime }$, reduced scattering coefficient; ${\mathrm{BF}}_{i}$, blood flow index.

Monte Carlo (MC) simulations were performed using Monte Carlo eXtreme^22^ in the MATLAB environment (Mathworks, Natick, MA, United States) on a standalone workstation (Ryzen 9 3800x, Corsair 64 GB RAM, and Nvidia RTX 2070). LUTs were generated as inverse models based on MC results. MC-based multi-layer DCS LUTs have been described in prior works^19^^,^^23^ and multi-layer FD-NIRS LUTs are an extension of single-layer LUTs that our group has previously demonstrated.^24^ Briefly, a semi-infinite geometry was defined with three layers: the skin, adipose, and muscle. A source and detector were positioned on top of the skin layer with a source–detector separation (SDS) of 25 mm [Fig. 1(b)]. Paired single-layer models were also developed for comparison [Fig. 1(a)]. The upper two layers (skin and adipose) of the multi-layer simulations were assigned static optical properties, and the skin layer was assigned a static thickness. Assumed values were informed by prior literature and are shown in Table 1. The skin optical properties were determined from a prior study that used spatial frequency domain imaging to measure skin optical properties and used the Fitzpatrick skin type scale to classify the subject’s skin tones.^25^ Subjects with a Fitzpatrick score of less than 16 were categorized as having light skin tone, subjects with a score of greater than 26 had dark skin tone, and subjects that scored between 16 and 26 had medium skin tone.^25^ In addition, prior literature has shown that upper subcutaneous tissue (skin + adipose) has a ${\mathrm{BF}}_{i}$ baseline value of $0.6\;{\mathrm{mm}}^{2}/s$. For each wavelength, the bottom layer was first assigned as $\mu_{a}=0\;{\mathrm{mm}}^{-1}$ and 10 simulations were run for each $\mu_{s}^{\prime }$ value with a defined step size of $0.1\;{\mathrm{mm}}^{-1}$ over a range of 0.1 to $1\;{\mathrm{mm}}^{-1}$. Photons were launched from the source position and recorded at the detector location. For each detected photon, the total pathlength and the total dimensionless momentum transfer were recorded.^23^ For DCS, both the total pathlength and total momentum transfer information were fed into the electric temporal field autocorrelation function equation^23^ alongside $\mu_{a}$ and ${\mathrm{BF}}_{i}$ to obtain the temporal field autocorrelation function ($G_{1}$). The resulting $G_{1}$ and an instrument-dependent coherence factor (0.45 for phantom measurements and ∼0.35 for *in vivo* measurements) were then input into the Siegert equation^33^ to obtain the normalized temporal autocorrelation function ($g_{2}$). In the case of FD-NIRS, the pathlength and $\mu_{a}$ were input into Beer’s Law to calculate absorption weights.^24^^,^^34^ The resulting photon absorption weights were then binned based on photon time of flights to create photon time-of-flight distributions. A Fourier transform was applied to the photon time-of-flight distributions function to generate a complex frequency-domain reflectance measurement at a given modulation frequency. This procedure was done for each MC simulation that had a distinct bottom layer $\mu_{s}^{\prime }$. Linear interpolation was performed on both the complex FD reflectance and $g_{2}$ from each distinct MC simulation to create high-density LUTs, the $\mu_{s}^{\prime }$ interpolation step size was 0.001 for the complex FD reflectance and 0.05 for the $g_{2}$. Each individual simulation took ∼5  min and a single LUT took on average 15 min to create. Fitting 16 min of data collected at a sampling frequency of 0.5 Hz took ∼25  s for either FD-NIRS or DCS dataset.

**Table 1:** Properties of the skin, adipose, and muscle layers and the corresponding references used in the MC simulations and $g_{2}$ calculations.

Layer	Thickness (mm)	$\mu_{a}$ (${\mathrm{mm}}^{-1}$)		$\mu_{s}^{\prime }$ (${\mathrm{mm}}^{-1}$)		g	n	${\mathrm{BF}}_{i}$ ($10^{-6}\;{\mathrm{mm}}^{2}/s$)	References
		730 nm	830 nm	730 nm	830 nm				
1. Light skin	1	0.019	0.006	1.34	1.17	0.9	1.40	0.6	9, 25–29
1. Medium skin	1	0.051	0.017	1.34	1.17	0.9	1.40	0.6	9, 25–29
1. Dark skin	1	0.149	0.044	1.34	1.17	0.9	1.40	0.6	9, 25–29
2. Adipose	1 to 6	0.010	0.010	1.19	1.09	0.9	1.44	0.6	9, 28, 30, 31
3. Muscle	93+	0.01 to 0.2	0.01 to 0.2	0.1 to 1.0	0.1 to 1.0	0.9	1.38	0.1 to 40	28, 32

### 2.2 Stackable Multi-Layer Silicone Phantoms

Stackable silicone phantoms were fabricated to validate the multilayer LUTs [Fig. 1(c)]. Thin silicone phantoms were created to mimic skin and adipose layers. These phantoms consisted of clear silicone (Eager Polymers, P4 Silicone), nigrosin (Sigma-Aldrich, N4754), and titanium dioxide (Sigma-Aldrich, 248576) and were cast in custom molds to ensure accurate layer thicknesses (1, 2, and 4 mm). The custom mold consisted of two clear acrylic pieces with one plate having an overflow hole and a 3D-printed perimeter piece with the desired thickness. A technical drawing of the mold is shown in Fig. S1 in the Supplementary Material. Thin phantoms were fabricated by pouring the silicone solution on the bottom plate and perimeter then laying the top plate, excess material flowed out from the overflow hole. The full phantom recipes are shown in Table S1 in the Supplementary Material. A matching larger bulk homogeneous phantom (70×108×38  mm) was made for each thin phantom and used to measure the optical properties using a custom FD-NIRS system. The measured optical properties are shown in Table 2. The thin phantoms were stacked to create a multi-layer phantom with the bottom layer being either a silicone phantom mimicking muscle optical properties for the FD-NIRS validation dataset or a liquid phantom for DCS validation. The liquid phantom consisted of intralipid (Fresenius Kabi, Intralipid 20%) and nigrosin. A custom DCS system was used to measure the ${\mathrm{BF}}_{i}$ from the Brownian motion of the liquid phantom. The liquid phantom’s recipe is shown in Table S1 in the Supplementary Material, and the optical properties and ${\mathrm{BF}}_{i}$ are shown in Table 2.

**Table 2:** The optical properties of silicone and intralipid phantoms. Values for g and n were assumed.

Phantom	Thickness (mm)	$\mu_{a}$ (${\mathrm{mm}}^{-1}$)		$\mu_{s}^{\prime }$ (${\mathrm{mm}}^{-1}$)		g	n	${\mathrm{BF}}_{i}$ ($10^{-6}\;{\mathrm{mm}}^{2}/s$)
		730 nm	830 nm	730 nm	830 nm			
Light skin	1	0.010	0.007	1.62	1.27	0.9	1.4	0
Medium skin	1	0.059	0.040	1.36	1.09	0.9	1.4	0
Dark skin	1	0.106	0.073	0.78	0.60	0.9	1.4	0
Adipose	1, 2, 4	0.008	0.006	1.49	1.27	0.9	1.4	0
Silicone muscle	30	0.041	0.072	0.42	0.38	0.9	1.4	0
Intralipid muscle	50	0.007	0.006	0.40	0.34	0.61	1.37	1.05

### 2.3 Sensitivity Analysis

Several assumptions related to the skin and adipose layers in the multi-layer models were made based on prior literature (Table 2). These include the skin thickness as well as the skin and adipose optical properties. Although assigning fixed values to the model parameters reduced computational complexity, any discrepancies between the assumed properties and measured tissue could induce errors in extracted target tissue optical properties. A sensitivity analysis was conducted to quantify how errors in these assumptions would affect measured target tissue properties. Each assumed model parameter was perturbed one at a time in separate MC simulations to evaluate the effect on extracted target tissue $\mu_{a}$, $\mu_{s}^{\prime }$, and BFi. The magnitude of the tested model perturbations is shown in Table 3 and is based on the reported variance of baseline literature values from healthy subjects. The perturbation percentages were defined as the approximate reported standard deviations divided by the mean of each parameter. The exception was the adipose thickness where the perturbation was set to 10%. Sensitivities were determined for the case of 1-mm adipose thickness and light, medium, and dark skin tones. Sensitivity for $\mu_{a}$, $\mu_{s}^{\prime }$, and ${\mathrm{BF}}_{i}$ was defined as

$$\text{Sensitivity}=\frac{\frac{\left(\text{Recovered Parameter}-\text{Ground Truth}\right)}{\text{Ground Truth}}\times 100\%}{\text{Perturbation}\;\%}.$$

**Table 3:** Perturbation values for the model assumptions and the corresponding reference that justifies the magnitude of the perturbation.

Perturbation type	Perturbation (%)	References
Skin thickness	10	26
Skin $\mu_{a}$	75	25
Skin $\mu_{s}^{\prime }$	25	25
Adipose thickness	10	—
Adipose $\mu_{a}$	30	30
Adipose $\mu_{s}^{\prime }$	30	30
Skin ${\mathrm{BF}}_{i}$	30	35
Adipose ${\mathrm{BF}}_{i}$	25	36

### 2.4 Healthy Volunteer Study

All measurements were conducted under an institutionally approved protocol (BU IRB 5618E), and all subjects completed a consent form prior to the experiments. A custom system that consisted of FD-NIRS (730 nm modulated at 139 MHz and 830 nm modulated at 149 MHz) and DCS (850 nm) was used to measure the muscle tissue of the sternocleidomastoid muscle during breathing exercises.^37^ The SDS was 25 mm. The study protocol is explained in detail in a previous work.^37^ Briefly, 17 healthy young (26.1±1.8 years) volunteers performed a breathing exercise that consisted of a baseline, load, and recovery period while the sternocleidomastoid, a neck muscle, was monitored. The load consisted of subjects breathing through a respiratory trainer and each subject performed both a low load and a high load. A colorimeter (PCE Instruments, PCE-CSM1) was used to determine the subject’s individual typology angle (ITA).^38^^,^^39^ The determined ITA was then used to classify each subject’s skin tone as either light (n=7), medium (n=6), or dark (n=4) according to the thresholds determined by literature. An ITA>41  deg was classified as light skin tone, an ITA<10  deg was classified as dark skin tone, and an ITA between 10 deg and 41 deg was classified as medium skin tone.^38^^,^^39^ In addition, an ultrasound (GE, Vscan Extend) with a broad-bandwidth linear array (3.4 to 8 MHz) was used to determine the adipose thickness (2.4±0.7  mm). The skin was assumed to be 1-mm thick for all subjects.

Recovered target tissue $\mu_{a}$ and ${\mathrm{BF}}_{i}$ values were processed to obtain chromophore values and the metabolic rate of oxygenation (${\mathrm{MRO}}_{2}$). Oxygenated hemoglobin plus myoglobin (oxy [Hb + Mb]) and deoxygenated hemoglobin plus myoglobin (deoxy [Hb + Mb]) concentrations were calculated using Beer’s Law from $\mu_{a}$ values at 730 and 830 nm with an assumption of 20% lipid fraction and 62.5% water fraction.^40^^–^^43^ Total hemoglobin plus myoglobin (total [Hb + Mb]) is the addition of oxy [Hb + Mb] and deoxy [Hb + Mb]. Tissue saturation ($S_{t}O_{2}$) is defined as

$$S_{t}O_{2}=\frac{\mathrm{Oxy}[\mathrm{Hb}+\mathrm{Mb}]}{\text{Total}[\mathrm{Hb}+\mathrm{Mb}]}\times 100\%.$$

The ${\mathrm{MRO}}_{2}$ is defined by the following equation:^9^

$${\mathrm{MRO}}_{2}=\mathrm{HGB}\times {\mathrm{BF}}_{i}\times \frac{S_{p}O_{2}-S_{t}O_{2}}{\text{venousratio}\times \mathrm{mw}\;\text{of}\;\mathrm{Hb}}.$$

The following assumptions were made in the ${\mathrm{MRO}}_{2}$ calculations: hemoglobin concentration of blood (HGB) values of 14 and 16  g/dL for females and males, respectively,^44^ a peripheral arterial oxygen saturation (${\mathrm{SpO}}_{2}$) of 98%,^45^ a blood volume fraction in venous compartments (venous ratio) of 0.75,^46^ and the molecular weight of hemoglobin (mw of Hb) of 64,500  g/mol.^44^

The Wilcoxon signed-rank test was used to evaluate the statistical significance of values derived from the single-layer versus multi-layer LUTs. The test was performed for all muscle parameters at both baseline and during the peak of the perturbation. Baseline values were calculated by finding the mean value of the first 50 s of the measurement. For the perturbation value, the largest absolute maximum difference from the baseline measured in either the low or the high load was selected as the perturbation value. Additional subanalyses were conducted to disentangle the effects of the skin tone and adipose thickness. The difference between the single layer and multi-layer for both baseline and perturbation were compared with both adipose thickness and skin tone. Robust linear regression analysis was conducted to show the strength of the relationship that each parameter difference had with skin tone and adipose thickness, respectively.

## 3 Results

### 3.1 Stackable Multi-Layer Silicone Phantoms

**Fig. 2:**
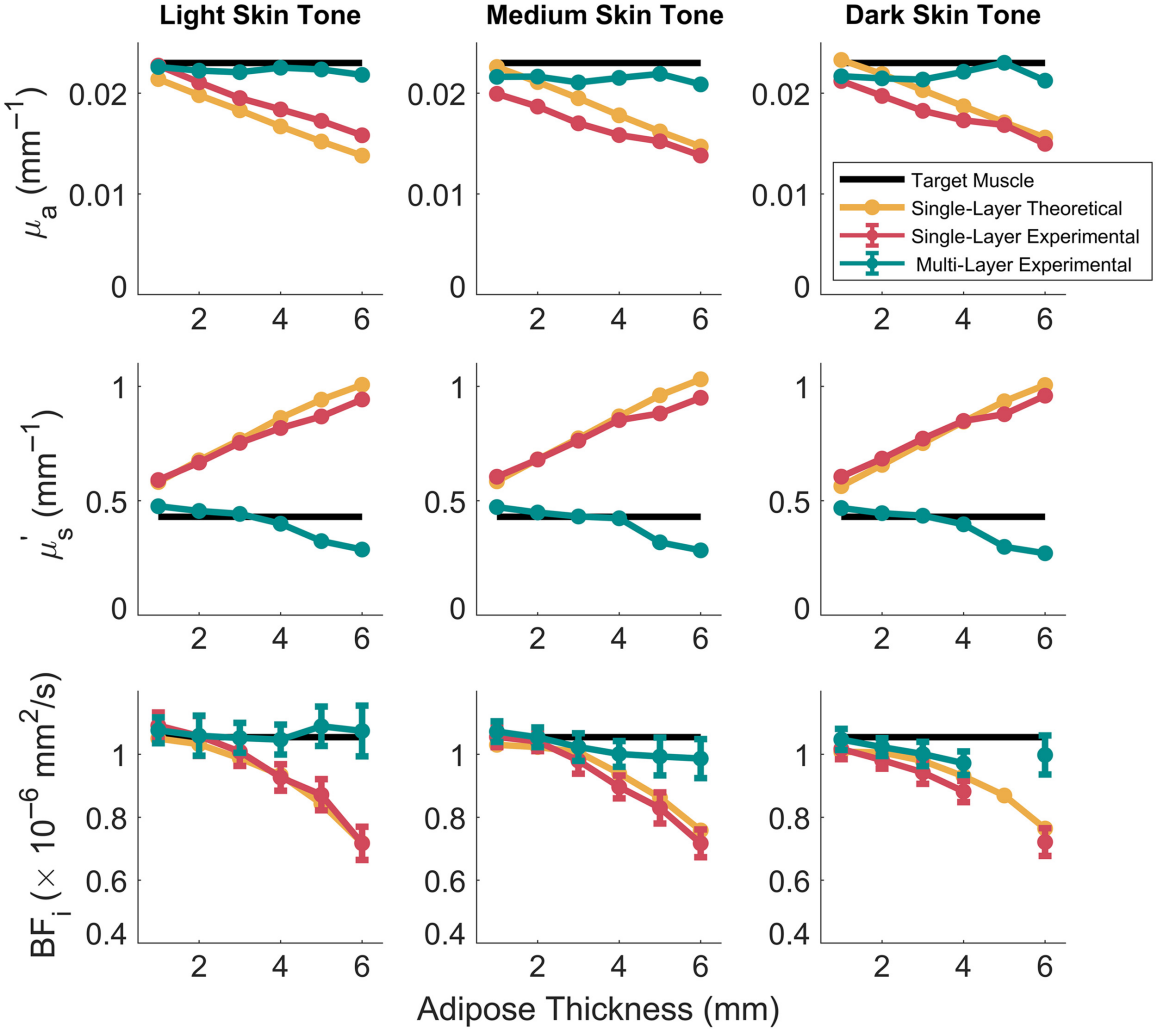
Recovered target $\mu_{a}$ and $\mu_{s}^{\prime }$ at 830 nm and ${\mathrm{BF}}_{i}$ from multi-layer phantom measurements. When measurements were processed with multi-layer LUTs, the recovered values (teal line) closely matched those of the known ground truth of the bottom layer (black line). In addition, the recovered values calculated via a single-layer LUT (red line) matched the expected extractions from simulations (yellow line). An experimental measurement error occurred for ${\mathrm{BF}}_{i}$ for the dark skin phantom at 5-mm adipose thickness, and those data are not shown. $\mu_{a}$, absorption coefficient; $\mu_{s}^{\prime }$, reduced scattering coefficient; ${\mathrm{BF}}_{i}$, blood flow index.

The multi-layer LUTs greatly improved the accuracy of target tissue optical properties in stackable silicone phantoms across skin tone and adipose thicknesses, as shown in Fig. 2. Here, the recovered $\mu_{a}$ and $\mu_{s}^{\prime }$ at 830 nm, as well as the ${\mathrm{BF}}_{i}$ are shown for both the simple single-layer LUTs (single-layer experimental values) and multi-layer LUTs (multi-layer experimental values). In addition, the known target tissue properties (target muscle values) are indicated as well as the expected extractions for the single-layer case from MC simulation (single-layer theoretical values). In general, when the mimicked skin tone and adipose thickness were not taken into account (i.e., when a single-layer LUT was used), there was an underestimation of both $\mu_{a}$ and ${\mathrm{BF}}_{i}$. This underestimation increased as a function of adipose thickness, with errors reaching −35±4% and −32±0% at 6-mm adipose thickness, respectively. In addition, $\mu_{s}^{\prime }$ was overestimated by up to 121±2% at 6-mm adipose thickness. When the multi-layer model was used, the extracted properties closely matched the known target values across all the skin tones and adipose thickness. Similar trends were seen in the recovered $\mu_{a}$ and $\mu_{s}^{\prime }$ at 730 nm, as shown in Fig. S2 in the Supplementary Material.

### 3.2 Sensitivity Analysis

**Fig. 3:**
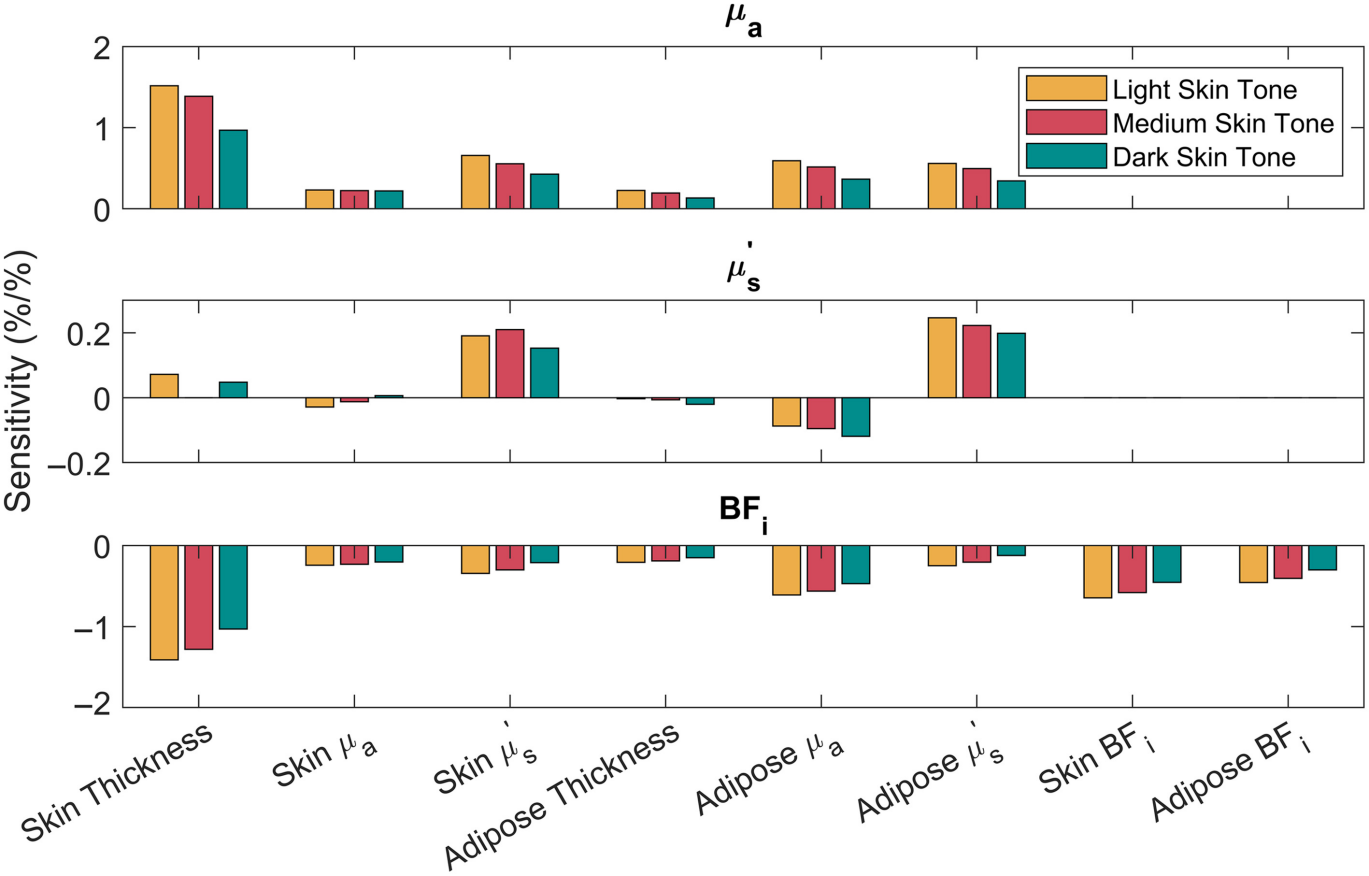
Sensitivity of the multi-layer model to different model assumptions for extractions of $\mu_{a}$ and $\mu_{s}^{\prime }$ at 830 nm, and ${\mathrm{BF}}_{i}$ model was most sensitive to skin thickness for extractions of both $\mu_{a}$ and ${\mathrm{BF}}_{i}$. No substantial differences were observed across skin tones. $\mu_{a}$, absorption coefficient; $\mu_{s}^{\prime }$, reduced scattering coefficient; ${\mathrm{BF}}_{i}$, blood flow index.

The sensitivity analysis results are shown in Fig. 3 for $\mu_{a}$ and $\mu_{s}^{\prime }$ at 830 nm and ${\mathrm{BF}}_{i}$. The sensitivities are presented as %/% (i.e., unitless) so that they can be compared across parameters. The model was most sensitive to the assumption of skin thickness, which had ∼1%/% sensitivity for $\mu_{a}$ and −1%/% for ${\mathrm{BF}}_{i}$. This means that a 1% mismatch in the skin thickness leads to a ±1% error in $\mu_{a}$ and ${\mathrm{BF}}_{i}$. Prior literature suggests that skin thickness can vary by ∼10% across different anatomic locations,^26^ which could lead to similar magnitude errors in $\mu_{a}$ and ${\mathrm{BF}}_{i}$. Although the sensitivity to skin $\mu_{a}$ was relatively low, approximately ±0.2%/%, this parameter is known to vary by as much as 75% across a measured population,^25^ which would lead to, for example, a ±15% error in target $\mu_{a}$. In general, $\mu_{s}^{\prime }$ was relatively insensitive to mismatches in model assumptions; the largest sensitivity was only ∼0.2%/% which arose from a perturbation in either skin or adipose $\mu_{s}^{\prime }$. Similar trends were seen for both $\mu_{a}$ and $\mu_{s}^{\prime }$ at 730 nm (Fig. S3 in the Supplementary Material). Although sensitivities had minimal dependence on the skin tone for the 830 nm results, this was not the case for 730 nm, in which the dark skin tone on average was less sensitive to model assumption compared with medium and light skin tones, especially for the $\mu_{a}$ results.

### 3.3 Healthy Volunteer Study

**Fig. 4:**
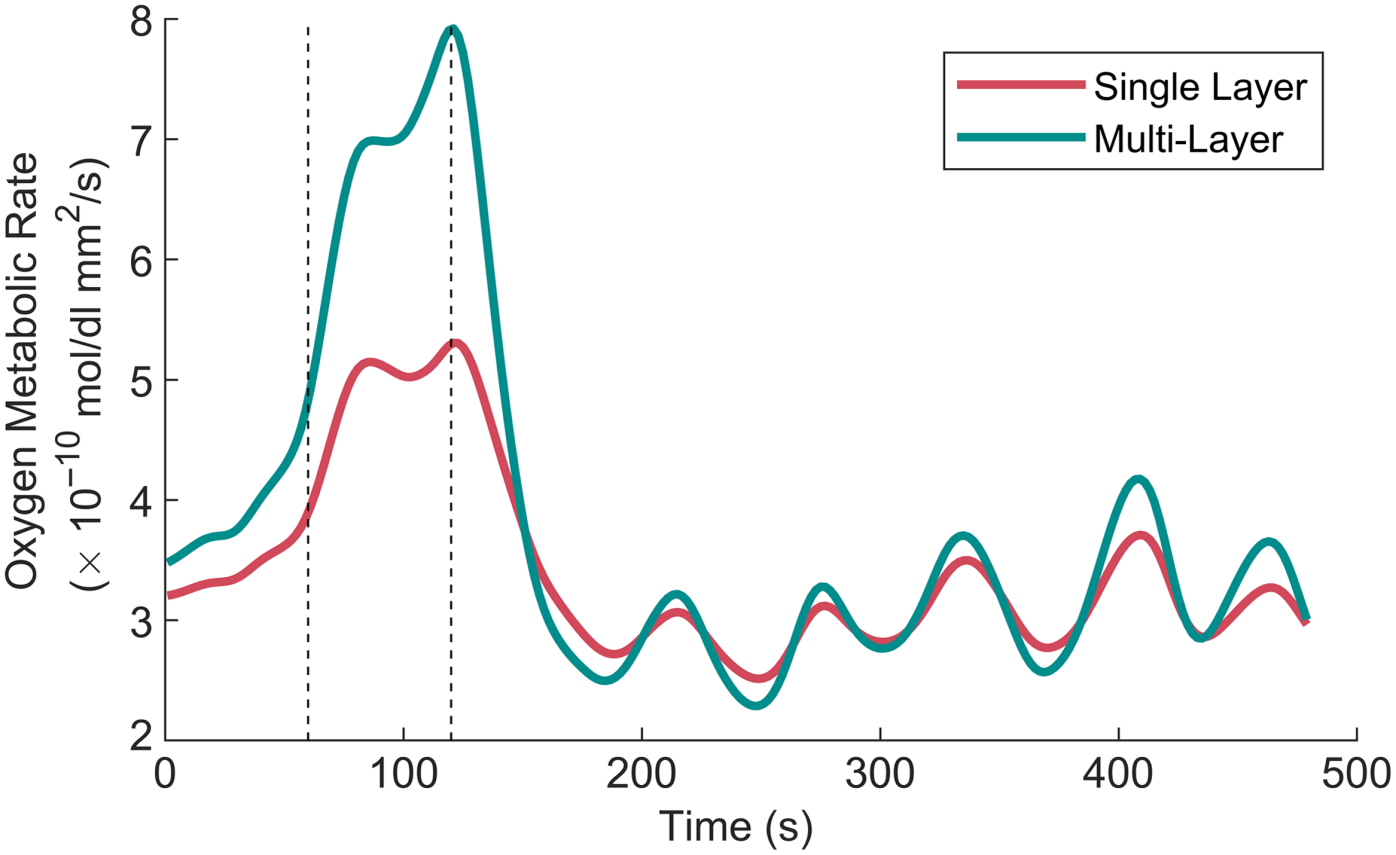
Example mean time traces for ${\mathrm{MRO}}_{2}$ from one subject with an ITA value of −17.8  deg (dark skin tone) and an adipose thickness of 1.5 mm. The values derived from single-layer LUTs are denoted by the red line, and the multi-layer LUTS values are denoted by the teal line. The vertical dashed lines indicate the start (t=60  s) and stop (t=120  s) of the loading phase. The ${\mathrm{MRO}}_{2}$ values were substantially underestimated when the simpler single-layer model was used.

**Fig. 5:**
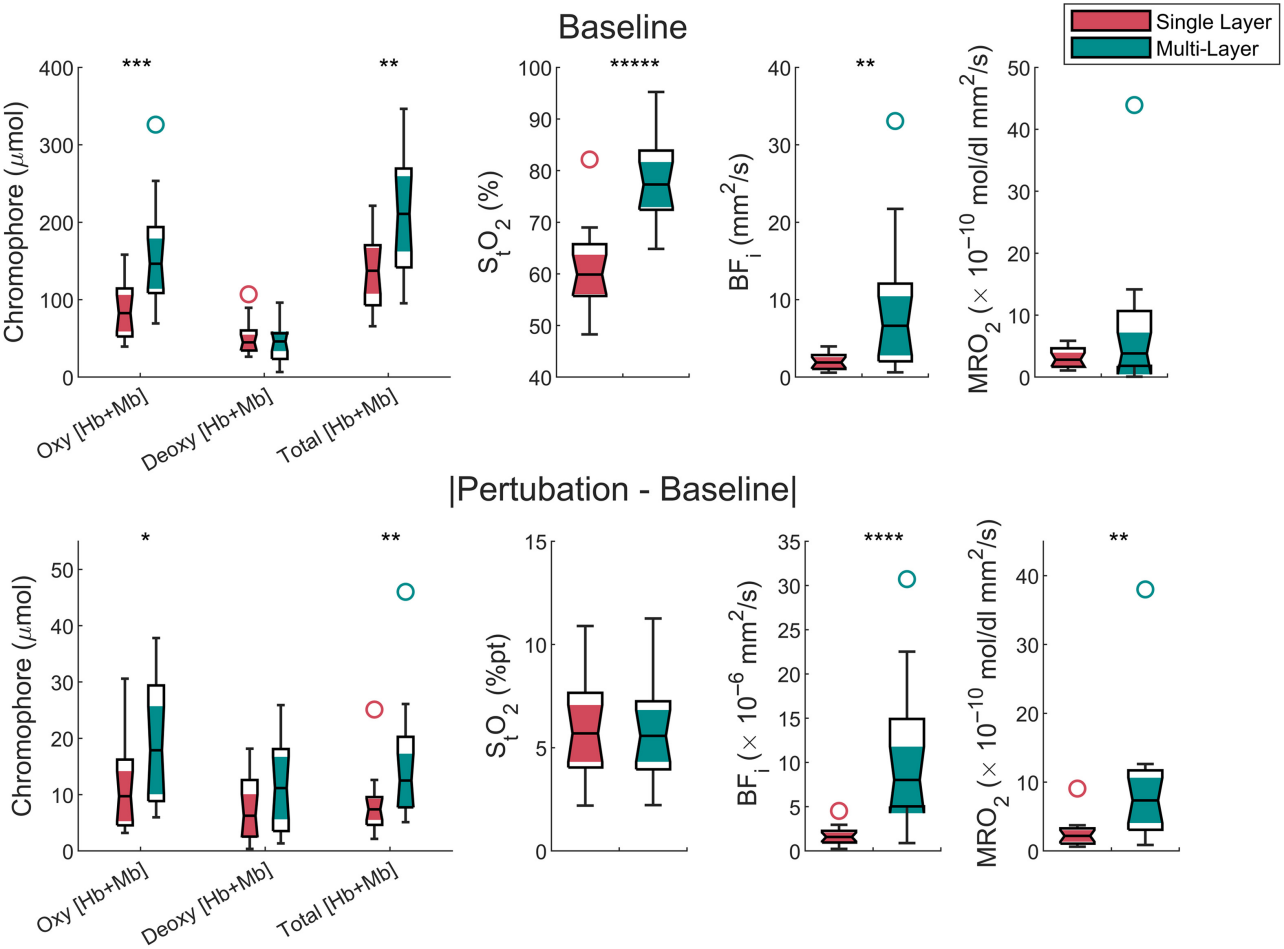
Comparison between single-layer and multi-layer values for both the baseline and the absolute difference of baseline values and the magnitude of perturbations during a breathing exercise. Baseline values overall were increased when a multi-layer LUT was used to recover the values except for deoxy [Hb + Mb] and ${\mathrm{MRO}}_{2}$. The perturbations from multi-layer LUT were larger than those from single-layer LUT except for deoxy [Hb + Mb] and $S_{t}O_{2}$. Each box chart displays the following information: the median which is denoted by the notch, the lower and upper quartiles which are denoted by the shaded area, any outliers which are denoted by circles, and the minimum and maximum values that are not outliers which are denoted by the whiskers. * p<0.05, ** p<0.01, *** p<0.001, **** p<0.0001, and ***** p<0.00001.

Example time traces for both single-layer and multi-layer results from a single subject are shown in Fig. 4, and others can be seen in Figs. S4 and S5 in the Supplementary Material. The time traces shown are those of ${\mathrm{MRO}}_{2}$, and the multi-layer derived values are more biologically plausible than the single-layer values. The comparison between *in vivo* measurements using the single-layer and multi-layer LUTs is shown in Fig. 5. There were statistical significances between extractions from the two models for both baseline and perturbation values. Notably, oxy [Hb + Mb], total [Hb + Mb], $S_{t}O_{2}$, and ${\mathrm{BF}}_{i}$ were underestimated with the single-layer model at baseline. Oxy [Hb + Mb] values were underestimated by 78±33  μmol, total [Hb + Mb] by 69±33  μmol, $S_{t}O_{2}$ by 18±9 %pt, and ${\mathrm{BF}}_{i}$ by $7\pm 8\times 10^{-6}\;{\mathrm{mm}}^{2}/s$. The perturbation magnitudes of oxy [Hb + Mb], total [Hb + Mb], ${\mathrm{BF}}_{i}$, and ${\mathrm{MRO}}_{2}$ were also underestimated with the single-layer model. Across most of the six muscle-based parameters for both the baseline and perturbation, the values from multi-layer LUTs had a wider distribution than the dataset derived from a single-layer model.

**Fig. 6:**
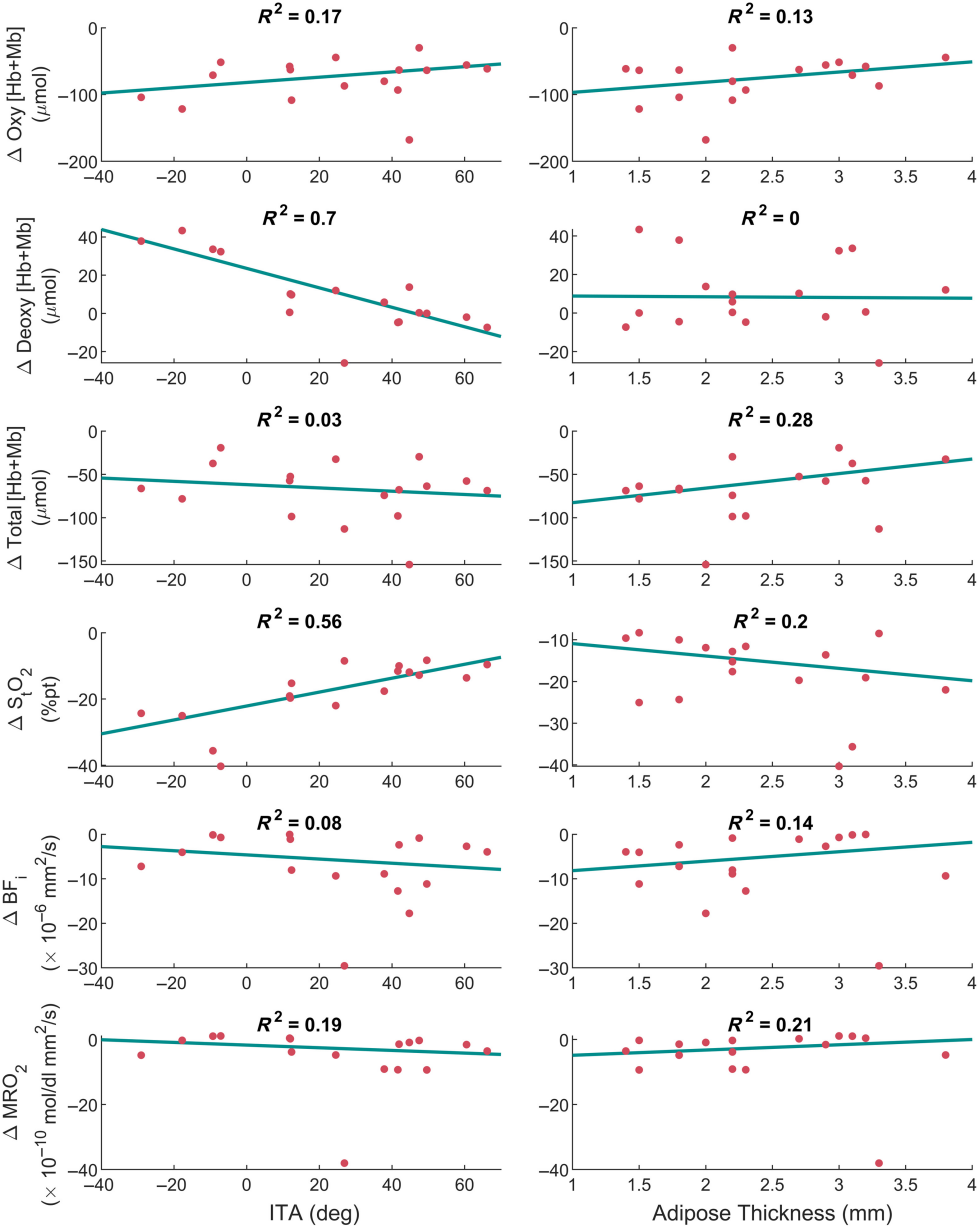
Relationship of skin tone (ITA) and adipose thickness on the differences in baseline optical parameter extractions between the models. Y-axis values show extractions from the multi-layer LUT subtracted from the single-layer LUT. These values can be interpreted as the extraction errors when using a single-layer LUT that does not take into account adipose thickness or skin tone. Therefore, a negative value means that the use of a simple single-layer model would cause an underestimate in that parameter compared with a multi-layer model. Robust linear regression analysis was used to determine the best-fit line and the $R^{2}$ value. The adipose thickness relationship with the six tissue parameters was relatively weak, $R^{2}<0.30$. Skin tone had stronger relationships, especially with deoxy [Hb + Mb] ($R^{2}=0.70$) and $S_{t}O_{2}$ ($R^{2}=0.56$).

The relationship between skin tone and adipose thickness and the difference in baseline values between the two LUTs are shown in Fig. 6. Adipose thickness had weak relationships for all six parameters, with all $R^{2}<0.30$. Skin tone was strongly correlated to both differences in extracted deoxy [Hb + Mb] and $S_{t}O_{2}$, both having an $R^{2}>0.50$. Skin tone and deoxy [Hb + Mb] had the strongest relationship and showed that the single-layer LUT overestimated deoxy [Hb + Mb] for dark skin tone subjects while underestimating the lighter skin tone subjects. $S_{t}O_{2}$ was underestimated for all skin tones, with the largest underestimates occurring for the darkest skin tones. There were no $R^{2}>0.2$ for the differences in perturbation magnitude (Fig. S6 in the Supplementary Material).

## 4 Discussion

In this study, we demonstrated that using subject-specific multi-layer inverse models that account for both skin tone and adipose thickness significantly impacted FD-NIRS and DCS results. Modeling and phantom studies showed that the multi-layer model enhanced the accuracy of extracted target tissue optical properties. Sensitivity analysis indicated that although the model was relatively insensitive to most assumptions, skin thickness was the most sensitive parameter, with inaccuracies potentially leading to errors in the extracted tissue’s $\mu_{a}$ and ${\mathrm{BF}}_{i}$. When applied to healthy volunteers, our results revealed that both baseline properties and perturbation magnitudes during respiratory exercises were underestimated by the simpler single-layer model that did not consider skin tone or adipose thickness. The underestimates in $S_{t}O_{2}$ could be quite large, as much as 20 %pt in some cases. Further analyses indicated that skin tone had a more substantial effect than adipose thickness on the extracted tissue properties, and tissue $S_{t}O_{2}$ was underestimated in subjects with darker skin tones unless a subject-specific multi-layer model was used.

Both modeling and phantom measurements revealed that the simple-single layer inverse model was highly influenced by the upper skin and adipose layers, and extractions tended toward these upper-layer optical properties, especially as the adipose thickness increased. This explains the underestimation in $\mu_{a}$ and ${\mathrm{BF}}_{i}$ as well as the overestimation in $\mu_{s}^{\prime }$ when the single-layer model was used to extract properties from the multi-layer phantoms. That is to say, the upper layers (skin and adipose) had lower $\mu_{a}$ and ${\mathrm{BF}}_{i}$ and a higher $\mu_{s}^{\prime }$ compared with the target bottom layer. For the *in vivo* data, the single-layer inverse model consistently underestimated both the baseline values and perturbations when compared with the recovered values from a multi-layer model for many of the extracted parameters. In addition, this effect in the baseline values was skin tone- and adipose thickness-dependent. Although there has been little prior work related to the effect of skin tone and adipose thickness for FD-NIRS and DCS, the general effect of surface-enhanced measurements has been previously characterized for similar technologies. For example, a prior MC-based sensitivity analysis on cerebral measurements showed that the scalp had a large effect on both continuous wave NIRS and DCS measurements.^47^ It was found that increasing the SDS provided a decreased sensitivity to the superficial scalp. In the case of a 5% perturbation in total [Hb + Mb] in the scalp, there was a sensitivity of 0.95, 0.88, and 0.88 for 10, 20, and 30 mm SDS.^47^ In addition, a 20% perturbation in blood flow of the scalp resulted in a sensitivity of 1.00, 0.94, and 0.80 for 10, 20, and 30 mm SDS. For this study, an SDS of 25 mm was utilized, and a longer SDS may further improve sensitivity to the target tissue layer at the expense of signal quality.

Prior work from different imaging modalities has shown that measurements are affected by both skin tone and adipose thickness.^13^^–^^15^^,^^48^ For example, measurements of peripheral arterial oxygen saturation ($S_{p}O_{2}$) measured with pulse oximetry have shown a bias toward overestimation of $S_{p}O_{2}$ in patients with darker skin, thus missing hypoxia in many cases.^13^ In a prior study focused on photoacoustic imaging (PAI) measurements between 700 and 900 nm, it was shown that the blood oxygen saturation (${\mathrm{sO}}_{2}$) was overestimated in simulations and in *in vivo* mouse measurements by as much as 9 %pt for dark skin tones. This was attributed to the increase in spectral coloring with darker skin tones.^14^ The opposite effect was observed in this study, where $S_{t}O_{2}$ was underestimated by as much as 20 %pt for subjects with dark skin tones. This difference likely arises from the fundamentally different imaging contrasts and measurement geometries employed by pulse oximetry, PAI, and FD-DOS. In the case of PAI, a superficial melanin layer decreases PA signal from underlying blood vessels at shorter wavelengths, which gets interpreted as an underabundance in deoxyhemoglobin and a subsequent overestimate of ${\mathrm{sO}}_{2}$. Conversely, for FD-DOS, the increase in optical attenuation caused by melanin may skew measurement sensitivity toward more superficial layers, including both the skin and adipose, which is interpreted as an underabundance of oxyhemoglobin and an overabundance of deoxyhemoglobin and thus a lower $S_{t}O_{2}$. This highlights the importance of understanding the context and modality-specific effects of the skin tone. In terms of adipose thickness, a prior MC modeling study showed that the PPG signal to noise ratio was decreased by as much as 62% for obese subjects.^15^ In this study, we observed that $S_{t}O_{2}$ underestimates were most severe for subjects with the thickest adipose layer. Together, these results highlight how both skin tone and adipose thickness optical parameter extractions.

There were several limitations to this study worth discussing. The first is that specific assumptions were made about the thickness and optical properties of the upper tissue layers. These values were derived from prior healthy volunteer studies and may not apply to different disease states or during perturbations. A potential way to reduce errors associated with these assumptions is to incorporate a short SDS in combination with a multi-layer inverse model to measure the upper layers alongside the deeper tissues.^9^^,^^49^ The second limitation was the relatively small healthy volunteer study size. There was a large variance in subject response from the respiratory exercise; this made it difficult to fully isolate the effects of skin tone and adipose thickness. The linear regression analysis shows skin tone- and adipose-dependent trends, but they were relatively weak overall ($R^{2}<0.30$). The strength of these trends could potentially be increased by a larger study population. In addition, these multi-layer improvements *in vivo* are only validated via the phantom measurements, but no ground truth was measured due to the invasive nature of these measurements. Other limitations include the fact that only three different categories of skin tone were utilized (light, medium, and dark), and a more continuous scale (i.e., ITA) might further improve the accuracy of results. In addition, only one kind of dynamic perturbation was tested, and only one anatomic site was measured. Results may vary for site to site and for the type of perturbation. Finally, the patient-specific inverse models require both an evaluation of the skin tone with a colorimeter, and adipose thickness with ultrasound, which necessitate additional equipment and measurements.

## 5 Conclusion

We have developed subject-specific multi-layer LUTs that take into account both skin tone and adipose thicknesses to recover muscle parameters more accurately from FD-NIRS and DCS. These multi-layer models were validated with multi-layered phantom experiments, and the results showed the ability to correctly recover underlying information accurately up to 7 mm of upper tissue (1-mm skin thickness + adipose thickness). Sensitivity analysis shows potential sources of error from assumed invariant upper layers of the models. The multi-layer LUTs were used to process human data, and analysis was done to compare a simple single-layer and subject-specific multi-layer LUTs. There were significant differences between both the baseline values and perturbation from various tissue parameters. Both skin tone and adipose thickness had a linear relationship with the inaccuracies that were accrued from the usage of a single-layer model for the majority of tissue parameters. Skin tone in particular increased the inaccuracies of deoxy [Hb + Mb] and $S_{t}O_{2}$. This study helps to highlight the importance of taking into account both skin tone and adipose thickness in FD-NIRS and DCS measurements.

## Supplementary Material

10.1117/1.BIOS.2.1.012503.s01

## Data Availability

Minimum datasets for phantom measurements, sensitivity analysis, and subject measurements are provided with the present article. Furthermore, scripts and associated instructions for the creation of LUTs are provided in a repository on Github: https://github.com/BU-BOTLab/LUTs_paper_BD2024. Further information can be provided upon request.
