# Supplementary material for: Effects of skin tone and adipose thickness on frequency domain near-infrared spectroscopy and diffuse correlation spectroscopy
Source: Biophotonics Discov. 2024 Dec 6;2(1):012503. doi: 10.1117/1.BIOS.2.1.012503 (PMC13098740; doi:10.1117/1.BIOS.2.1.012503)
Supplement: Supplementary file 1 [file BIOS_002_012503_SD001.pdf]

Supplementary Information

Table S1: Phantom recipes

Phantom	Nigrosin (mg/L)	TiO_2 (g/L)	Intralipid (%)
Light Skin	3.2	1.6	-
Medium Skin	12.2	1.6	-
Dark Skin	20.2	1.1	-
Adipose	3.0	1.6	-
Silicone Muscle	9.1	1.3	-
Intralipid Muscle	4.0	-	0.34

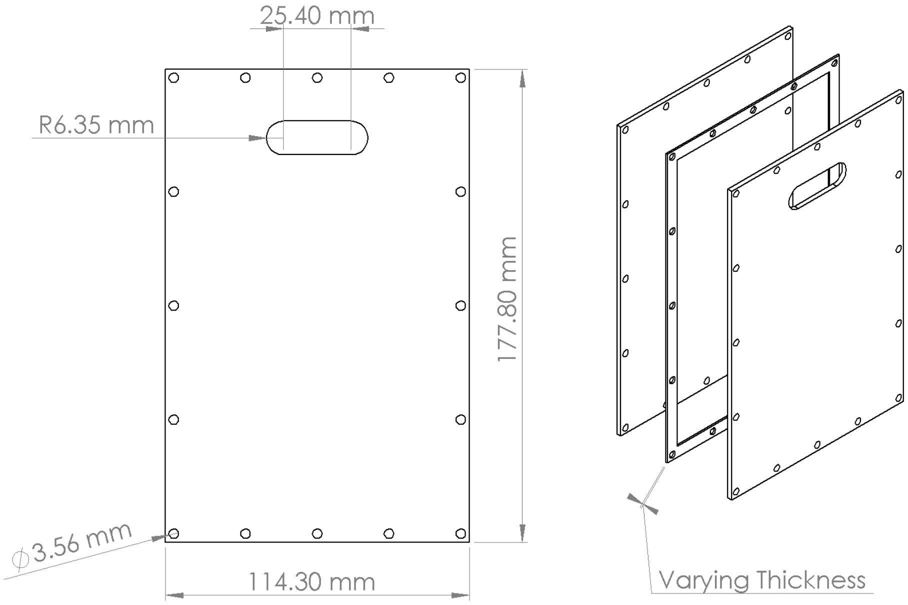

Figure S1: Drawing of custom made mold for thin stackable phantoms. Mold consists of three components: a bottom acrylic plate, a 3D printed perimeter with desired thickness, and a top acrylic plate with an overflow hole.

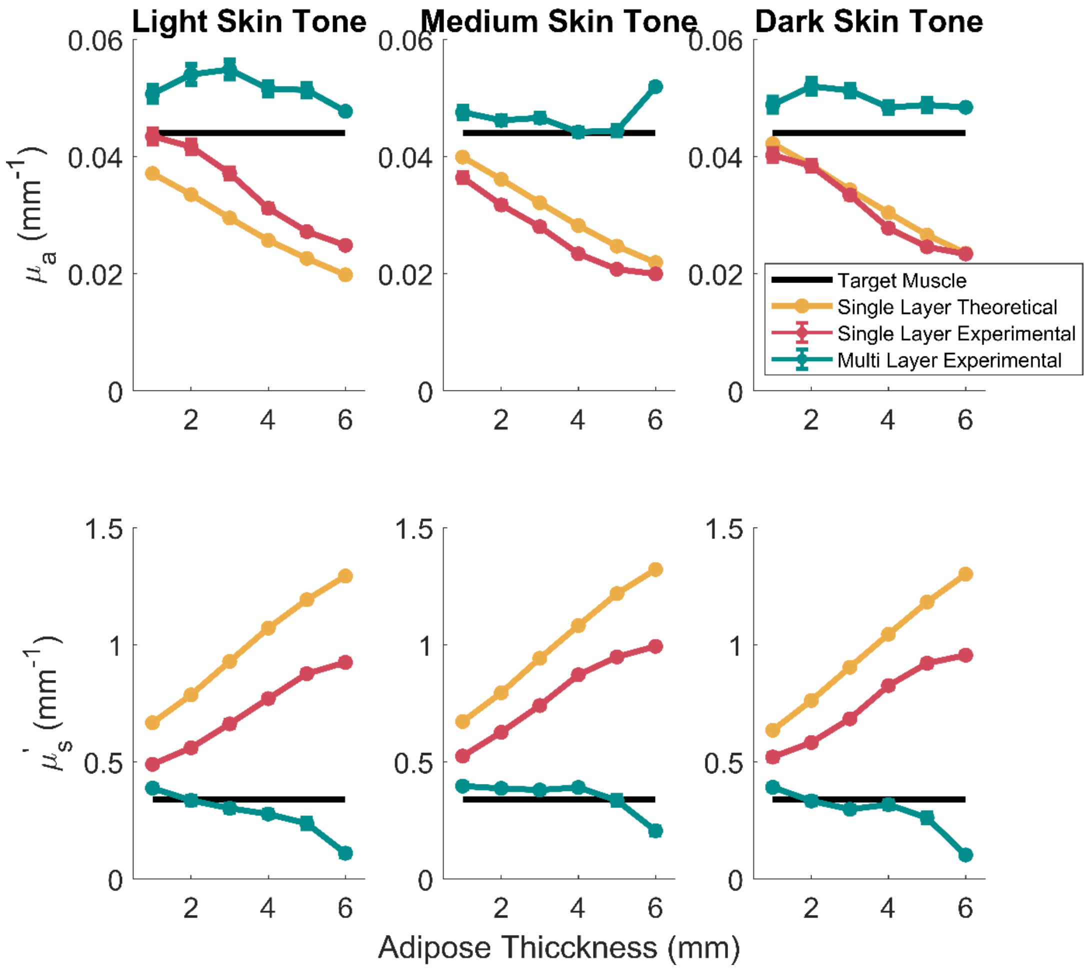

Figure S2: Recovered bottom layer's μ_a at 730 nm and μ_s' at 730 nm from multi-layer phantom measurements and expected errors.

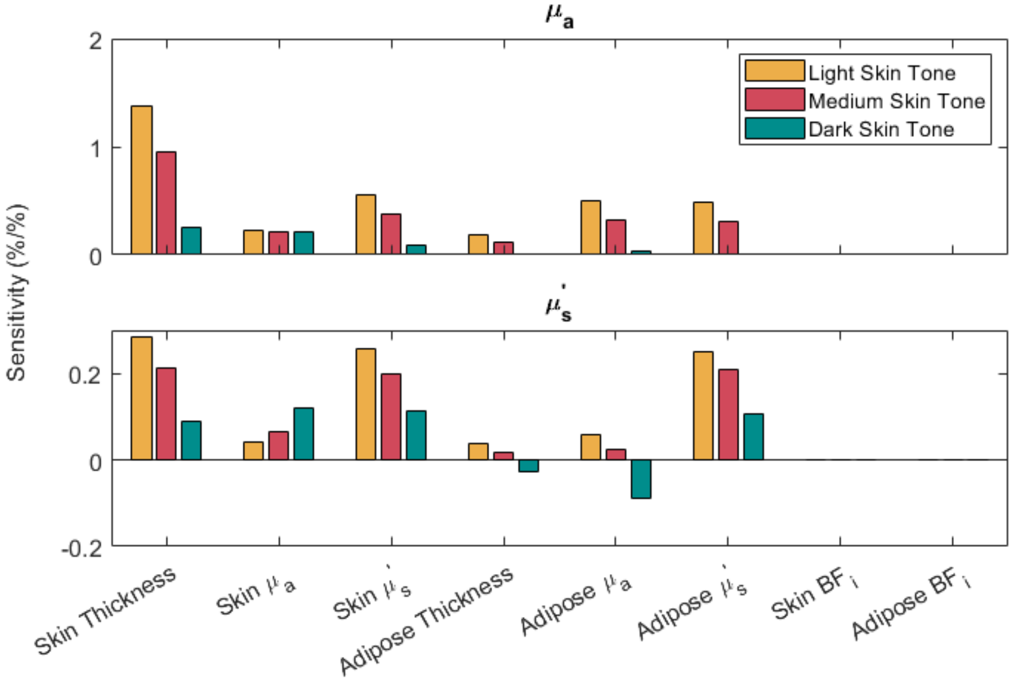

Figure S3: Sensitivity analysis for μ_a at 730 nm and μ_s' at 730 nm for eight different perturbations.

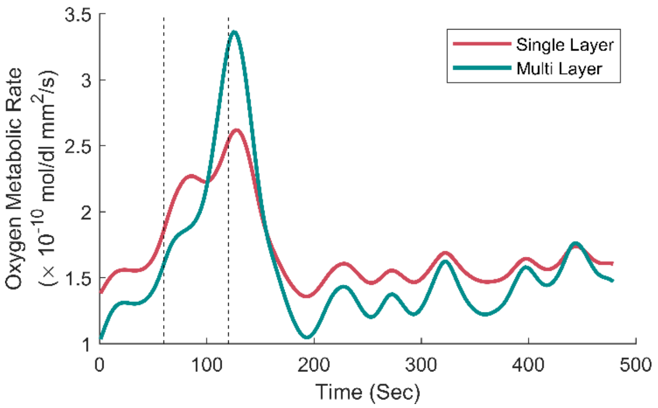

Figure S4: Example mean time traces for MRO_2 from one subject with an ITA value of 12.01˚ (medium skin tone) and an adipose thickness of 2.7 mm. The values derived from single layer LUTs are denoted by the red line and the multi-layer LUTS values are denoted by the teal line. The vertical dashed lines indicate the start (t = 60 secs) and stop (t = 120 sec) of the loading phase.

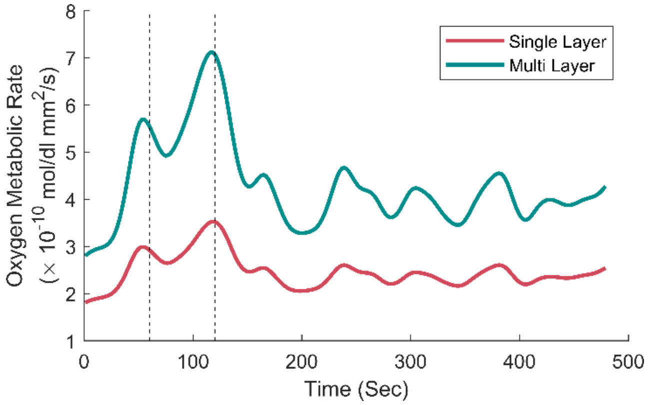

Figure S5: Example mean time traces for MRO_2 from one subject with an ITA value of 41.94˚ (light skin tone) and an adipose thickness of 1.8 mm. The values derived from single layer LUTs are denoted by the red line and the multi-layer LUTS values are denoted by the teal line. The vertical dashed lines indicate the start (t = 60 secs) and stop (t = 120 sec) of the loading phase.

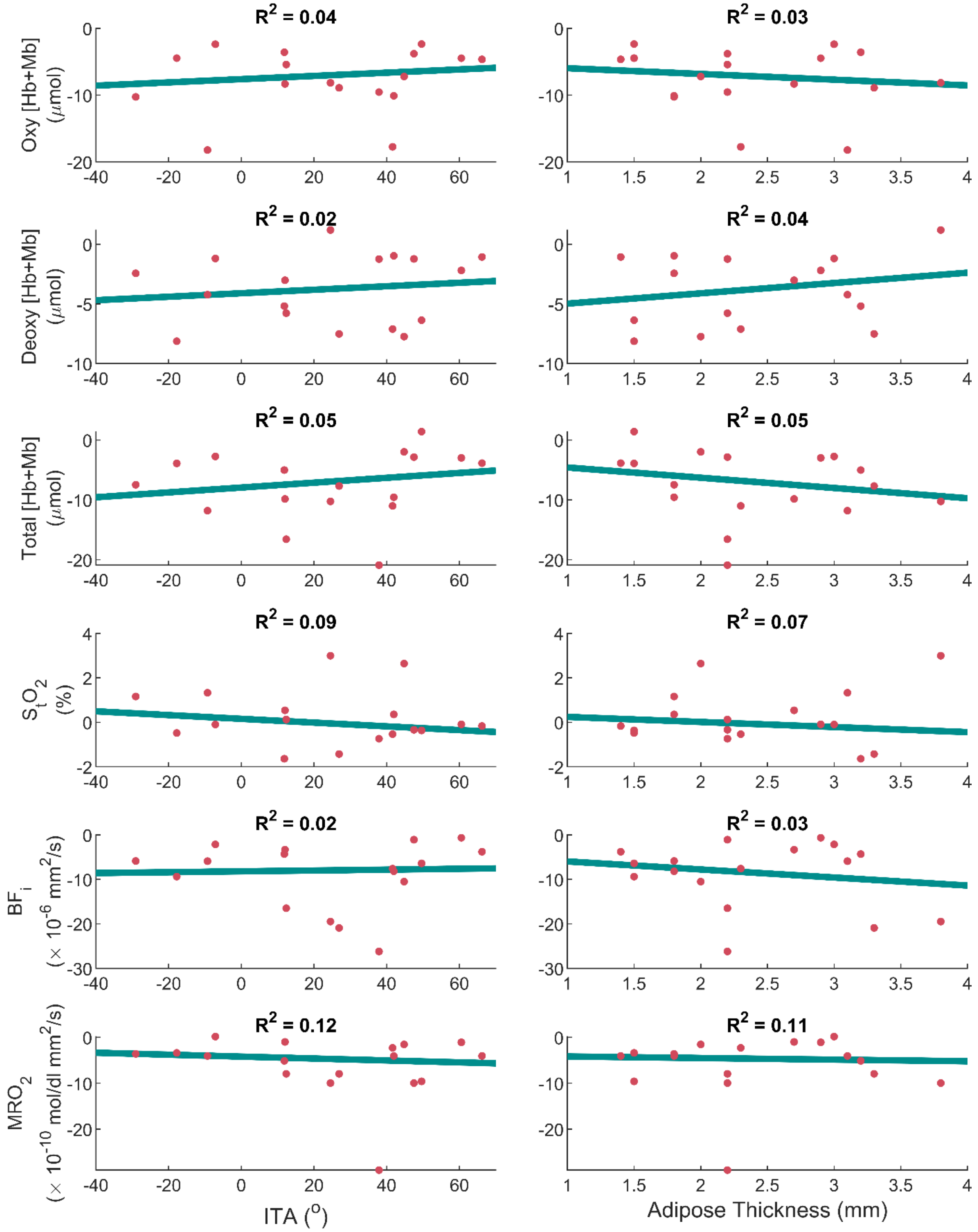

Figure S6: The robust linear regression analysis of the difference in pertubation values between the two LUTs vs skin tone (ITA) and adipose thickness.
